# A Homozygous Frameshift Mutation in *GREB1* Leads to Female Infertility

**DOI:** 10.1155/humu/8849136

**Published:** 2026-05-06

**Authors:** Jinwei Yang, Linyan Yang, Bo Yan, Hongbao Li, Lin Zhang, Ting Hu, Xingyi Chao, Chuan Zhang, Yali Ni, Zhiqiang Wang

**Affiliations:** ^1^ Reproductive Medical Center, Gansu Provincial Maternity and Child-Care Hospital (Gansu Province Central Hospital), Lanzhou, Gansu, China; ^2^ Medical Genetics, Gansu University of Traditional Chinese Medicine, Lanzhou, Gansu, China, gszy.edu.cn; ^3^ Department of Physiology and Pathophysiology, School of Basic Medical Sciences, Xi′an Jiaotong University, Xi′an, Shaanxi, China, xjtu.edu.cn; ^4^ Gansu Province Medical Genetics Center, Gansu Provincial Maternity and Child-Care Hospital (Gansu Province Central Hospital), Lanzhou, Gansu, China

**Keywords:** female infertility, *GREB1*, mutation, whole-exome sequencing

## Abstract

Primary infertility affects 15% of couples worldwide, yet many genetic causes remain unknown. Through whole‐exome sequencing of a woman with primary infertility and repeated embryo implantation failure, we identified a novel homozygous frameshift mutation in *GREB1* (c.5364delC, p.Ala1789Argfs∗42). Sanger sequencing confirmed heterozygous carrier status in both parents. Functional studies demonstrated the absence of nonsense‐mediated mRNA decay but abnormal *GREB1* protein expression via western blotting. Computational modeling predicted pathogenic structural alterations. This variant is absent in gnomAD/ExAC databases. Our findings establish *GREB1* mutations as a novel cause of female infertility, highlighting its role in endometrial receptivity regulation.

## 1. Introduction

Infertility in couples of childbearing age has become common, possibly due to environmental and lifestyle factors [1]. Infertility affects more than 15% of couples worldwide. Infertility can adversely affect the physical and psychological well‐being of both individuals and families [2]. The World Health Organization defines infertility as a failure to conceive after 12 months or more of regular sex without contraception [3]. About 85% of infertile couples have a clearly defined etiology. Common causes include female tubal disease, endometrial dysfunction, ovulation disorders, reduced ovarian reserve, insufficient endometrial decidualization, and male oligospermia, azoospermia, or abnormal sperm morphology [8, 7, 6, 4, 5]. The etiology of infertility is extremely complex, and some causes are still unknown. It is currently believed that about 15% of the pathogenic factors may be hereditary. Genetic variations involving reproductive function are likely to affect the development of the reproductive organs. Such variations may lead to disorders of gamete formation and development, as well as reduce the receptivity of the female endometrium. Consequently, embryo implantation may fail, ultimately affecting pregnancy outcomes [10, 9].

To date, a number of genes, including *TUBB8*, *WEE2*, *PATL2*, *PLCZ1*, *NLRP2*, and *NLRP5*, have been shown to be related to oocyte maturation, fertilization, and embryonic development [2, 3, 11, 12, 13]. It is likely, given the complexity of the reproductive system, that there are many more potential pathogenic genes and variants. Recently, *GREB1*, located on Chromosome 2, was identified in breast cancer cells [14]. This gene is closely involved in the estrogen and progesterone signaling pathways and is expressed in estrogen‐dependent tissues and organs, such as the breast [15], ovary [16], and endometrium [10]. The expression of *GREB1* is increased significantly under estrogen stimulation, and the encoded protein is involved in regulating cell proliferation, differentiation, and migration, all of which are important in reproductive health. However, it is not known whether *GREB1* variants are associated with infertility, nor whether the gene is involved in the regulation of reproductive function.

In the present study, using whole‐exome sequencing and functional analyses, we identified a homozygous *GREB1* mutation (c.5364delC, p.A1789Rfs∗42) in a woman with primary infertility, suggesting a possible pathogenic role of this variant in infertility.

## 2. Materials and Methods

The study was conducted in accordance with the principles of the Declaration of Helsinki and was approved by the Ethics Committee of the Maternal and Child Health Hospital of Gansu Province (Gansu Provincial Central Hospital) with Ethical No. 2023GSFYLS78. All subjects participating in the study and their legal guardians provided written informed consent.

### 2.1. Patients

A couple consulted the Reproductive Medicine Center of Gansu Provincial Maternal and Child‐care Hospital (Gansu Provincial Central Hospital) in 2024 after failing to conceive despite not having used contraception for 6 years. The woman was 28 years old, 155 cm tall, and weighed 52 kg. The woman′s menstrual cycle is irregular, the menstrual cycle is 1–6 months, and the rest of the gynecological examinations were normal. The man was aged 32 years, with a height of 175 cm and a weight of 63 kg. His semen parameters were essentially normal with a concentration of 62.73 × 10^6^/mL, a forward movement sperm ratio of 55%, and a normal morphology rate of 4.6%. The chromosomal karyotypes of the husband and wife were normal, and primary infertility could thus only be diagnosed clinically according to the existing test results. For this reason, the couple was treated with assisted reproductive technology twice, fresh cycle transplantation once, and frozen‐thawed embryo transplantation once, all of which resulted in a failure to conceive.

### 2.2. Genetic Analysis

Five milliliters of peripheral blood was collected from both the husband and wife, and genomic DNA was extracted for genetic analysis. High‐throughput sequencing was used for detection. In addition, specific methods, such as qPCR and capillary electrophoresis, were also employed to detect mutations that might have been missed by NGS. The data were analyzed using bioinformatics, and the sequencing results were aligned with the human genome hg19 (GRCh37) reference sequence. Potential mutation sites and frequencies were investigated by searching the human exome database ExAC (http://exac.broadinstitute.org/) and the genomic variation frequency database gnomAD (http://gnomad.broadinstitute.org/). New variants were examined using the Human Gene Mutation Database (HGMD; http://www.hgmd.cf.ac.uk/ac/) and the ClinVar database (http://www.ncbi.nlm.nih.gov/clinvar/). Bioinformatics software, such as PolyPhen‐2 (http://genetics.bwh.harvard.edu/pph2/), MutationTaster (https:///www.mutationtastter.org/), and SWISS‐MODEL (https://swissmodel.expasy.org/interactive), were used to evaluate the potential pathogenicity of the variants. InterVar (http://wintervar.wglab.org/) software was also used to assess mutation pathogenicity according to the standards and guidelines of the American College of Medical Genetics and Genomics (ACMG) ([17]). Sanger sequencing was also performed on the woman′s parents to verify variations in candidate genes and determine their inheritance patterns.

### 2.3. Cell Culture, Expression Vector Construction, and Transfection

The identified variant in *GREB1* was a frameshift variant located before the last 50 bp of the penultimate exon of the gene. It was speculated that the pathogenicity of this mutation site may have been caused by nonsense‐mediated mRNA decay (NMD). As the expression of *GREB1* is very low in peripheral blood, to verify this conjecture, a minigene plasmid, pcDNA3.1(+), was constructed to verify mRNA degradation by transfecting 293T cells (NMD‐EJC model). The plasmid included the Exon 30–33 region. For the minigene assays, 293T cells (density, 2–3 × 10^5^) were seeded in 35 cm^2^ plates in 2 mL of 10% minimum essential medium (MEM) and grown overnight. Cells were transfected with DNA (4 *μ*g per well) using Lipofectamine 3000 (Thermo Fisher, Waltham, Massachusetts, United States) for 48 h and were then harvested by trypsinization. Total RNA was isolated using TRIzol reagent (Thermo Fisher) and phenol–chloroform extraction. The RNA was reverse‐transcribed to cDNA using the HiScript II 1st Strand cDNA Synthesis Kit (+gDNA wiper) (Vazyme, Nanjing, China). The cDNA was amplified using qPCR; information on the primers is provided in Table 1.

**Table 1:** Sequences of primers used for qPCR.

Primer name	Sequence 5^′^ − 3^′^	Target gene
*GREB1*‐qPCR‐F	ACGCTGGCTATCCCTATGAC	*GREB1*
*GREB1*‐qPCR‐R	CTCATTGTCCACTCGGCTAC	
*TUBB*‐F	TTAGAACCAACCAGGTGCTGAA	*TUBB* (internal reference)
*TUBB*‐R	GATGGGAAATGGACATACTTAGAAA	

### 2.4. Western Blotting

Total proteins were extracted from endometrial cells of the patient and normal women using RIPA buffer. Proteins (10 *μ*g) were separated on SDS‐PAGE gels and transferred to polyvinylidene difluoride (PVDF) membranes. The membranes were blocked with 5% BSA in TBST buffer at room temperature for 1 h and then incubated with a primary antibody against *GREB1* overnight at 4°C, followed by incubation with the corresponding secondary antibodies at a dilution of 1:5000 for 1 h at room temperature. After three washes (10 min each), the bands were visualized with chemiluminescence and imaged using an ImageQuant LAS 4000 mini chemiluminescence CCD camera (GE Healthcare Life Sciences, United States).

## 3. Results

### 3.1. Clinical Characteristics of the Affected Individuals

The couple received two cycles of controlled ovarian stimulation during in vitro fertilization (IVF) embryo transfer. The first cycle adopted a long‐acting long‐term protocol in the follicular phase, using Gn12 days, E2: 2916 pg/mL, and P: 0.52 ng/mL. Twelve eggs were obtained, with 11 mature oocytes in the MII stage, 1 oocyte in the GV stage, and 2 double pronucleus fertilized eggs (2PN) after fertilization by intracytoplasmic sperm injection (ICSI). One was a two‐cell embryo; seven mature oocytes were not fertilized. On the 3rd day of culture, the embryos were evaluated according to the Chinese expert consensus on morphological evaluation of human cleavage stage embryos and blastocysts [18]. One six‐cell Grade II embryo could be used on the 3rd day. The endometrial thickness was 12 mm, and luteal support after transplantation was provided by administration of progesterone soft capsules (Approval Number H20130336; specification, 0.1 g: 10), external use, two capsules per dose, three times a day, and dydrogesterone (Approval Number H20170221; specification, 10 mg), with 20 mg given orally twice a day. After 14 days of continuous use of all drugs, blood *β*‐HCG tests indicated no pregnancy. The second cycle used the progestin‐primed ovarian stimulation (PPOS) protocol, with Gn12 days, E2: 3333 pg/mL, and P: 0.69 ng/mL, resulting in 11 eggs, all of which were mature oocytes. After ICSI fertilization, five mature oocytes formed double pronucleus fertilized eggs (2PN), with two abnormal fertilized eggs (1PN and 3PN), while the remaining four were unfertilized (0PN). Four available embryos (two eight‐cell Grade II embryos, one nine‐cell Grade II embryo, and one six‐cell Grade II embryo) were cryopreserved on Day 3, and the frozen‐thawed embryos were selected for transplantation. The first frozen‐thawed embryo transfer used a downregulated hormone replacement cycle, consisting of injection of a long‐acting GnRH agonist on the 2nd day of the menstrual cycle for pituitary downregulation, and the size of the uterus was evaluated at 3–4 weeks after injection. After 4 weeks, estrogen and Femoston (Approval Number: H20150346; specification: 1 mg: 10 mg × 28) were given orally at a dose of 3 mg twice a day. After 7 days, B‐ultrasound was used every 3–5 days to monitor the thickness of the endometrium. After 11 days of medication, the endometrial thickness was only 6 mm, with E2 values of 1378 pg/mL and P of 0.15 ng/mL, so the transplantation was canceled. The second frozen‐thawed embryo transfer used a hormone replacement cycle: Estrogen was used from the 2nd day of the menstrual cycle, with 3 mg Femoston given orally twice a day. Seven days later, B‐ultrasound was used to monitor the intima thickness every 3–5 days, while adding Femoston vaginal medication, 1 mg once a day, with continual use for 7 days. After 14 days of treatment, the intima thickness was 9 mm, with E2 levels of 2033 pg/mL and P of 0.16 ng/mL. Intramuscular injections of 40 mg of progesterone (Chinese medicine Approval Number H33020828; specification: 1 mL: 20 mg) were administered once a day for 3 days after the frozen‐thawed embryo transfer involving the transplantation of two eight‐cell Grade II high‐quality embryos. The drugs used for luteal support after transplantation were Femoston (Approval Number: H20150346; specification: 1 mg:10 mg × 28) white tablets, oral medication: 1 mg twice a day; Femoston (Approval No.: H20150346; specification: 1 mg:10 mg × 28) gray tablets, oral medication: 2 mg twice a day; Femoston (Approval No.: H20150346; specification: 1 mg:10 mg × 28) white tablets, vaginal medication: 1 mg once a day; and snow ketone (Approval Number: H20140552; specification: 8% [90 mg]/branch), with 90 mg given once a day as a vaginal medication. All drugs were given for 14 days, after which the results of the blood *β*‐HCG test indicated no pregnancy.

### 3.2. Genetic Analysis

**Figure 1:**
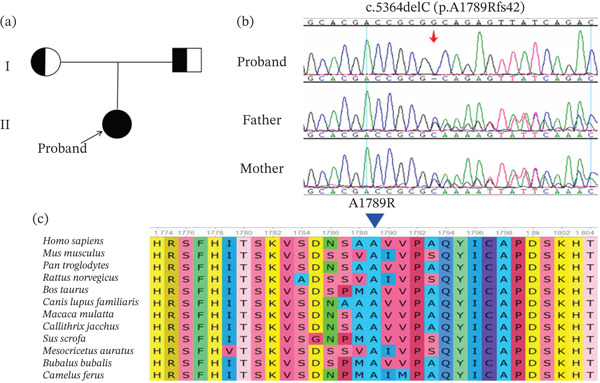
Genetic data and molecular findings of patients carrying *GREB1* variants. (a) Family pedigrees of the patient with the *GREB1* variant. (b) Sanger sequence validated *GREB1* variants. (c) Conservative analysis of variation sites across species (Alanine 1789th is highly conserved in different species, marked by a blue arrow).

**Figure 2:**
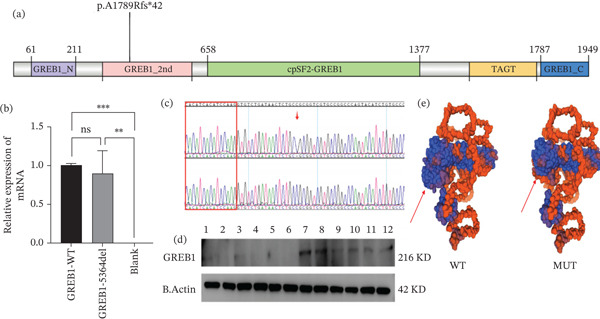
Functional characterization of the *GREB1* c.5364delC (p.A1789Rfs∗42) mutation. (a) Domain distribution of the *GREB1* gene variation. (b) mRNA expression levels in wild‐type and mutant cells. (c) cDNA *GREB1* gene c.5364delC (p. A1789Rfs∗42) locus sequencing in endometrial tissues of patients and normal controls. The arrow indicates the location of the mutation site, and the red box indicates the Exon 30 region. (d) WB test results: *GREB1* protein was almost not expressed in patients (Lanes 1–6 were patients; Lanes 7–12 were normal controls). (e) Three‐dimensional structure prediction of *GREB1* WT and MUT proteins. The double asterisk (^*^^*^) indicates that there is a statistically significant difference between GREB1‐5364del and the blank control group (*p* < 0.01).

Whole‐exome sequencing was performed on the couple. An unreported homozygous frameshift variant, c.5364delC (p.A1789Rfs∗42), in the *GREB1* gene was identified in the female patient. Sanger sequencing was performed on her parents, showing that both parents carried this heterozygous variant (Figure 1a,b). The variant occurred in Exon 31 and was predicted to cause the replacement of alanine at Position 1789 in the protein with arginine, followed by a frameshift and premature introduction of a stop codon, resulting in premature termination of the protein coding. This variant is rare and is not included in the general East Asian population in the gnomAD database. Functional analysis using bioinformatics indicated that Ala1789 was strongly conserved in different species (Figure 1c). This variant site occurs in the second structural domain of the *GREB1* protein (Figure 2a).

### 3.3. mRNA Degradation Caused by the Homozygous Frameshift Mutation in GREB1

The construction of the wild‐type and mutant *GREB1* minigene eukaryotic expression vectors, their transfection into 293T cells, and the extraction of the RNA, followed by reverse transcription into cDNA and amplification of the cDNA, showed no difference between the mRNA levels of the wild type and mutant (Figure 2b), indicating that the mutant did not develop NMD compared with the wild type.

To confirm that the c.5364del variant of *GREB1* did not lead to NMD, endometrial tissue was collected from the patient during the proliferative phase of the menstrual cycle to verify the pathogenicity of this locus. Normal volunteers were also recruited for a controlled trial. RNA was extracted from the endometrial tissue after sampling. After reverse transcription, the cDNA was amplified and sequenced. The primer sequences were *GREB1*‐F: 5 ^′^‐GTGCAGTACAACCAACCAGAACCG‐3 ^′^ and *GREB1*‐R: 5 ^′^‐GAGGAACGTGTGCTTGCTG‐3 ^′^. The PCR amplification of cDNA was consistent with the normal control band, and Sanger sequencing results indicated an absence of NMD (Figure 2c).

### 3.4. Western Blotting

To evaluate the effect of the mutation on *GREB1* protein expression, proteins were extracted from the endometrial tissues of the patient and normal women for WB analysis. The results showed that protein *GREB1* expression was abnormal in the patient (Figure 2d). An exploration of the relationship between phenotype and genotype indicated that the affected domain was involved in the *α*/*β* folding of the secondary structure of the protein and that the variant was likely to affect the function of this domain (Figure 2e). It is speculated that although this site does not cause NMD, the function of the *GREB1* protein may change due to premature termination of translation. According to the ACMG guidelines, the variant was classified as pathogenic (PVS1 + PM2_Supporting + PM3_Supporting).

## 4. Discussion

The *GREB1* gene is located on Chromosome 2p25.1, with a total length of about 108 kb and consists of 33 exons (chr2:11777859, NM_014668.3). *GREB1* has three different subtypes, namely, *GREB1a*, *GREB1b*, and *GREB1c*. *GREB1a* is a full‐length subtype, which produces *GREB1b* and *GREB1c* by alternative splicing and the introduction of premature stop codons in Exons 10 and 9, respectively [14]. At present, details of the structures and functions of the three *GREB1* subtypes remain largely unknown. The present study identified a homozygous frameshift mutation in the *GREB1* gene, which may lead to a decrease in female fertility. To date, there are no reports of this variant in the gnomAD database, and this study may expand the variation spectrum of infertility‐associated genes.

*GREB1* plays an important role in hormone‐responsive tissues. Analysis of the 10 kb region of the *GREB1* transcription start site has shown that it is regulated by various hormone receptors in the nucleus, such as the estrogen, progesterone, androgen, and glucocorticoid receptors [19]. It is widely recognized that the hypothalamus–pituitary–ovarian axis regulates the periodic secretion of estrogen and progesterone by the ovary. Estrogen promotes the proliferation of endometrial epithelial cells. Ovulation induces the secretion of progesterone and promotes the proliferation of stromal cells. Decidualization occurs during the secretory phase of the menstrual cycle. At this time, if there has been no successful fertilization of the mature oocytes, the thickened endometrium is shed, resulting in menstruation [20, 21]. Inappropriate responses of the endometrium to estrogen and progesterone can lead to infertility or abortion [23, 22].

In this study, before the first cycle of frozen‐thawed embryo transfer, during the process of endometrial preparation, and 11 days after estrogen administration, the transfer was canceled due to the thinness of the endometrium. This clinical phenotype indicates that the sensitivity of the endometrium to estrogen may be reduced by the *GREB1* mutation, with a poor response to low‐dose estrogen, leading to decreased ability of the endometrial epithelial cells to regenerate; it is not easy to achieve transplantable endometrial thickness under the action of exogenous low‐dose estrogen. In addition, a fresh cycle and freeze‐thaw cycle embryo transfer were also performed. Due to the high levels of exogenous estrogen used in these two cycles, an endometrial thickness suitable for transplantation was achieved, but no pregnancy resulted. This suggests that the *GREB1* mutation may adversely affect endometrial decidualization, thereby reducing endometrial receptivity and ultimately leading to implantation failure.

To further investigate the effects of the mutation, a *GREB1* minigene eukaryotic gene expression vector was constructed. After transfection, it was found that the mutation did not affect nonsense‐mediated mRNA degradation. In addition, endometrial samples from the patient and normal women were sequenced, and RT‐PCR analysis verified that the mutation did not lead to nonsense‐mediated mRNA degradation. However, western blotting showed that the *GREB1* protein expression in the patient was abnormal. It is therefore predicted that although this site does not cause nonsense‐mediated mRNA degradation, due to premature termination of translation, it may lead to changes in the function of the *GREB1* protein, resulting in a weakened endometrial response to estrogen and progesterone, impaired endometrial receptivity, and ultimately infertility.

In the menstrual cycles of normal women, *GREB1* expression is consistent with fluctuations in estrogen levels [24]. Estrogen levels are higher during follicular maturation and the midluteal phase, and *GREB1* levels are correspondingly higher than during the early follicular stage associated when the estrogen levels are low. The successful implantation of embryos requires the proliferation and differentiation of endometrial epithelial and stromal cells under the action of estrogen and progesterone [25]. At present, the physiological mechanisms regulating *GREB1* in the normal endometrium are not clear. A study by Chadchan et al. [26] showed that knockout of *GREB1* in female homozygous mice impaired endometrial decidualization, accompanied by low fertility and small litter sizes. Women with repeated implantation failures showed reduced expression of *GREB1* mRNA in the endometrium compared with normal women, while in women with endometriosis, *GREB1* expression was elevated in both epithelial and stromal cells [27]. A study by Camden et al. [9] demonstrated that *GREB1* is a progesterone‐responsive gene in both the mouse and human endometrium, and revealed that it can induce expression of *WNT4* and *FOXO1A*, key signaling molecules that control the fate of progesterone‐dependent endometrial decidualized cells. These studies have shown that *GREB1* plays an important role in the endometrium of women of childbearing age, and suggest that it may mediate the proliferation and differentiation of endometrial cells, although there is as yet no evidence of this.

This study also has some limitations. Specifically, functional analyses in cells and mice were not conducted. Studies will be undertaken in the future to verify the function of this mutation and elucidate the pathogenic mechanism associated with *GREB1* in infertility.

## 5. Conclusion

In summary, this study identified a novel pathogenic mutation in the *GREB1* gene in female patients with primary infertility using whole‐exome sequencing. The mutation is predicted to result in an abnormal protein structure that affects the proliferation and differentiation of endometrial cells. These findings provide a reference for the clinical diagnosis and genetic counseling of infertility.

## Author Contributions

**Jinwei Yang**: writing—review and editing, writing—original draft; **Linyan Yang**, **Bo Yan**, and **Hongbao Li**: writing—review and editing, conceptualization, supervision; **Lin Zhang**, **Ting Hu**, **Xingyi Chao**, and **Chuan Zhang**: conceptualization, supervision; **Yali Ni** and **Zhiqiang Wang**: supervision, writing—review and editing, funding acquisition, conceptualization. **Jinwei Yang**, **Linyan Yang**, and **Bo Yan** contributed equally to this work.

## Funding

This work was supported by the Natural Science Foundation of Gansu Province (23JRRA1389), the Gansu Province Science and Technology Project (25JRRA1235), the Gansu Province Key Research and Development Project (25YFFA056), and the Lanzhou Science and Technology Plan Project (2023‐2‐23 and 2024‐4‐30).

## Disclosure

All authors consent to the publication of this study. All the authors read and approved the final draft.

## Conflicts of Interest

The authors declare no conflicts of interest.

## Data Availability

The data that support the findings of this study are available from the corresponding authors upon reasonable request.
